# How long does it take to start minimal enteral feeding in preterm Neonates admitted to NICUs in Southern Oromia, Ethiopia?

**DOI:** 10.1186/s13052-025-01876-1

**Published:** 2025-02-07

**Authors:** Anteneh Fikrie, Terefu Yambo, Alo Edin, Miesa Gelchu, Dejene Hailu, Mark Spigt

**Affiliations:** 1 School of Public Health, Institute of Health, Bule Hora University, P.O. Box 144, Bule Hora, Ethiopia; 2 Department of Family Medicine, School for Public Health and Primary Care (CAPHRI), Care and Public Health Research Institute, Maastricht University, P.O. Box 616, Peter Debyeplein 1, 6229 HA Maastricht, Maastricht, 6200 MD Maastricht the Netherlands; 3 School of Public Health, College of Medicine and Health Sciences, Hawassa University, Hawassa, Ethiopia; 4 Department of Community Medicine, General Practice Research Unit, UiT the Arctic University of Norway, Tromsø, Norway

**Keywords:** Preterm neonates, Early trophic feeding, Predictors, Survival analysis

## Abstract

**Background:**

The timely initiation of trophic feeding (TF) is crucial for premature newborns, but challenging due to immaturity, respiratory instability, abdominal distension, resource scarcity, and healthcare worker expertise. Moreover, there is a dearth of information on predictors of full trophic feeding time. Therefore, this retrospective cohort study aimed to investigate the time it takes and its predictors to initiate minimal enteral feeding in preterm neonates in Southern Oromia, Ethiopia.

**Method:**

A facility-based retrospective follow up study was conducted among 434 randomly selected preterm neonates admitted to NICU of Bule Hora University Teaching Hospital and Yabello General Hospital from January 1, 2021 to December 30, 2022. Data were extracted by a pretested structured checklist, entered into Epidata 3.1 and then transferred to Stata version 17 for analysis. Kaplan Meier survival curve and log rank test were used to estimate survival time and a statistical comparison respectively. Bivariable and multivariable cox proportional hazard model was fitted to identify predictors of time to initiate TF and their outputs are presented using Adjusted Hazard Ratio (AHR) with 95% Confidence Intervals (CIs).

**Result:**

The overall incidence density of TF initiation was reported as 43.6 per 100 neonate-days. Moreover, the median (IQR) time to initiate TF was found to be 2 (1–4) days. Neonates delivered vaginally had a higher likelihood of early TF initiation (AHR: 1.64, CI: 1.26, 2.13), while those born between 32 and 34 weeks (AHR: 0.61, CI: 0.46, 0.81), VLBW neonates (AHR: 0.45, CI: 0.34, 0.60), neonates without KMC (AHR: 0.59, CI: 0.46, 0.79), and those in level II hospitals were less likely to start TF promptly (AHR: 0.78, CI: 0.62, 0.99). Furthermore, neonates with sepsis (AHR: 1.76, CI: 1.36, 2.28) and hypothermia (AHR: 1.51, CI: 1.19, 1.93) had delayed TF initiation.

**Conclusion:**

We observed a significant low rate of early TF initiation and higher death rate of preterm newborn in our study as compared to the global. Preterm neonates with lower GA, no KMC, and a VLBW are more likely to have a delayed initiation. Our results highlight that staff training on identifying neonates suitable for TF, and ensuring adequate resources for KMC in all NICU levels should be considered.

**Supplementary Information:**

The online version contains supplementary material available at 10.1186/s13052-025-01876-1.

## Background

According to World Health Organization (WHO), preterm birth is a birth before 37 completed weeks of gestation [1]. It is a significant global public health concern, accounting for nearly 1 in 10 babies born worldwide [2]. In 2019, 15.22 million neonatal preterm birth incidents occurred globally, with prevalence in Sub-Saharan Africa ranging from 3.4% to 49.4% [3]. In Ethiopia the prevalence ranges from 10.48–11.4% [4, 5]. Prematurity increases risks for short- and long-term complications due to underdeveloped gut mobility, microbiome, blood flow, and immunity [6–8]. Preterm neonates, with immature immune systems, face increased infection risk due to delayed or inappropriate feeding, which disrupts gut microbiota, necessitating timely and adequate feeding for growth and development [9, 10]. Trophic feeding (TF) also called minimal enteral nutrition, refers to the provision of enteral nutrition without the expectation of full nutritional needs being met, and it is the standard of care in neonatology for preterm neonates in the Neonatal Intensive Care Unit (NICU) [11, 12]. TF has a positive effect on growth, mortality and common morbidities among preterm neonates [13, 14]. Around 90% and 40% of preterm and very low birth weight (VLBW) infants experience growth delay due to inadequate enteral feeding initiation [8].

Clinical guidelines suggest the volume of feeding considered trophic for preterm newborns is 10–15 mL/kg/day and should be started within 24–48 h of birth [11], but implementation varies globally [15–17]. The European Society of Paediatric Gastroenterology, Hepatology and Nutrition (ESPGHAN) guidelines suggest starting with minimal enteral feeding of 20–25 ml/kg/day at 0.5–1 ml/kg/h, increasing by 20–25% every 24–48 h [18]. The Ethiopian NICU treatment guideline suggests early (within 24 h.) enteral feeding for preterm neonates [19], however initiating on time remains a challenge [20, 21]. A systematic review and meta-analysis of relevant study revealed early TF initiated within 96 h of birth, with volumes up to 24 ml/kg/day, reduces Necrotizing Enterocolitis (NEC) risk, potentially lowers late-onset sepsis, and improves gut maturation [22]. Numerous systematic reviews and meta-analyses claims early TF initiation in high-risk infants is safe and leads to improved neonatal feeding tolerance, maintenance of intestinal function during starvation, prevention of intestinal bacterial overgrowth and long-term outcomes for preterm infants [14, 23, 24].

Delayed initiation of TF is a global concern in NICUs and has been identified as a significant independent predictor of poor growth among preterm neonates [25]. It has been associated with increasing the risk of nutritional deficits, impaired brain growth, sepsis, feeding intolerance, low weight and poor survival [7, 24, 26]. Studies show significant delays and a median time of 7–13 days for full enteral feeding [27–29]. In Africa, only small proportions of premature infants start minimal feeding early [30–32]. Similarly, studies from Ethiopia show significant delays in TF for neonates, with 80–90% not receiving it within 48 h, 20% receiving it within 24 h, 29% dying before discharge, and 86.2% experiencing extra uterine growth restriction [21, 33, 34]. Therefore further determination of predictors of TF initiation is crucial for successful modification, development, and implementation of appropriate feeding guidelines for premature infants and improving clinical practice [20, 32, 35].

Although, previous studies identified several predictors of delayed initiation of minimal enteral feeding in preterm neonates, including physiological immaturity of the gastrointestinal (GI) tract, gestational age, maternal hypertension, cesarean delivery, respiratory distress syndrome, perinatal asphyxia, limited resources and healthcare worker expertise [24, 29, 36, 37], they had utilized relatively small sample sizes, and varied definitions of delayed initiation, making it challenging to compare and generalize the findings across different populations [30]. Moreover, according to a study, bettering prenatal and newborn care in remote hospitals improves the health of mothers and their children [38]. As a result, the current study considered the impact of NICU practices and protocols between two resource limited hospitals that were not consistently examined in previous research, Bule Hora University Teaching Hospital and Yabello General Hospitals, both located in peripheral areas and potentially marginalized.

## Methods and materials

### Study design and period

A two-year retrospective cohort study was conducted at public health facilities, with a study period covering January 1, 2021, to December 30, 2022. Data extraction for the study was performed between June 21 and July 18, 2023.

### Study area

The study was conducted in Bule Hora University Teaching Hospital (BHUTH) and Yabello General Hospital (YGH), Southern Oromia region’s largest hospitals. BHUTH located in Bule Hora town, offers various services to 5 million people of West Guji Zone and its surroundings. It has a total of 346 administrative and technical staffs. The services include pediatrics, emergency, delivery, outpatient, inpatient, laboratory, pharmacy, medicine and surgery. The NICU of the hospital has an approximate of 600 preterm neonate admissions per year [39]. On the other hand, Yabello General Hospital found in Yabello town, provides health services to 926,690 people in the Borena Zone, with an estimated 400 preterm neonate admissions annually in its NICU [40].

### Population, sample size determination and Sampling technique

All preterm neonates admitted to the NICUs of Bule Hora University Teaching Hospital and Yabello General Hospital during the specified timeframe (January 1, 2021—December 30, 2022) were eligible for our study. Records of neonates with incomplete charts, major congenital anomalies that interfere with feeding, those who developed NEC before initiation of enteral feeding and those who started breast feeding or TF prior to admission were excluded. We calculated the **s**ample size using Stata version 17 by considering AHR of 0.74 [21] two-sided 5% significant level, power of 80% and 20% considered for incompleteness of the data. Then minimum sample size obtained was 476. The total sample size was proportionally allocated to each study hospital and systematic random sampling technique was used to select study subjects from the list of medical records. Sampling interval (k) was calculated by dividing total population at each hospital for sample size for respective hospitals (k = 2). So, the 2nd medical record number was randomly selected from the sampling frame.

### Data abstraction tool and quality management

The study used a data abstraction checklist to ensure data quality, with a pretest conducted on 5% of preterm neonates at Adola General Hospital. Based on the pretest result, educational level of the mother was removed whereas kangaroo mother care and antibiotic were added to the checklist. The data were extracted by experienced four bachelors of science nurse professionals, with supervision and daily evaluations to ensure completeness and consistency. One-day training was given for data extractors and supervisors regarding data extractions.

### Study variables and operational definitions

Our study outcome variable was time to initiate TF, which was determined by subtracting the birth date from the date of TF initiation. Independent variables were neonatal related factors such as birth weight, sex, gestational age, hypothermia, meconium aspiration syndrome, first minute Apgar score, fifth minute Apgar score, perinatal asphyxia, sepsis, jaundice, hemodynamic instabilities and respiratory distress syndrome; maternal socio-demographic, medical and obstetric related factors such as age of the mother, residence, educational status, having antenatal care (ANC) follow up, premature rupture of membrane (PROM), preeclampsia, mode of delivery, postpartum hemorrhage, place of delivery, parity, gravidity and birth type and health service related factors such as kangaroo mother care, antibiotics, continuous positive air pressure, frequency of order revision and hospital type were used as independent variables.

#### Trophic feeding

The first minimal enteral feeding to prime the gut regardless of method [11, 41].

#### Early trophic feeding

A small volume of milk (less than 24 ml/kg/day) provided within 24–48 h of birth to stimulate gastrointestinal motility [14].

#### Survival time

The length of time in days followed starting from birth to the first trophic feeding or censorship.

#### Event

Initiation of first trophic feeding for preterm neonates.

#### Censored

Those neonates, who died, transferred or referred before starting trophic feeding or not started at end of follow-up.

#### Follow up time

Time from birth up to either the study subjects start trophic feeding or censored.

#### Hemodynamic instabilities

Patent ductus arteriosus, blood group and RH incompatibility, anemia, polycythemia, bleeding disorders, blood glucose disturbances [42].

### Data processing and analysis

Data were entered to Epi-Data version 3.1 and transferred to STATA version 17 for analysis. Descriptive statistics like frequency and percentage were used to describe the socio-demographic characteristics of the mother and the neonate. Continuous data containing variables were summarized with median and interquartile range. The Kaplan Meier survival curve and a log-rank test were used to estimate survival time and compare the survival curves of categorical variables respectively. To assess the validity of the Cox proportional hazard model, we checked its assumptions using Schoenfeld residual tests and − ln(− ln) survival plots (see Supplementary Table 1 and Supplementary Figs. 1a-e). The − ln(− ln) survival plots showed parallelism for all included covariates, indicating fulfillment of the proportional hazard assumption. Kaplan–Meier and predicted survival plots were also similar for all covariates (see Supplementary Figs. 2a-f). A bi-variable Cox-regression was computed for each predictor variable and a *p-*value of < 0.25 were used as cut off point to select variables to be entered into multivariable cox-regression. Accordingly, result of the final model was expressed in terms of adjusted hazard ratio (AHR) with 95% confidence interval and significant association was declared with a *p-*value less than 0.05 in a multivariable Cox regression model. Finally, result of the study is presented with tables, graphs, and text narrations.

## Results

From a total of 476 randomly selected records of preterm neonates, the data of 411 with complete medical records were extracted with a retrieval rate of 86.34%. Charts of 65 neonates were excluded (41 were due to initiation of breast feeding directly before TF, 20 were incomplete and 4 had congenital anomaly).

### Maternal clinical and obstetric characteristics

**Fig. 1:**
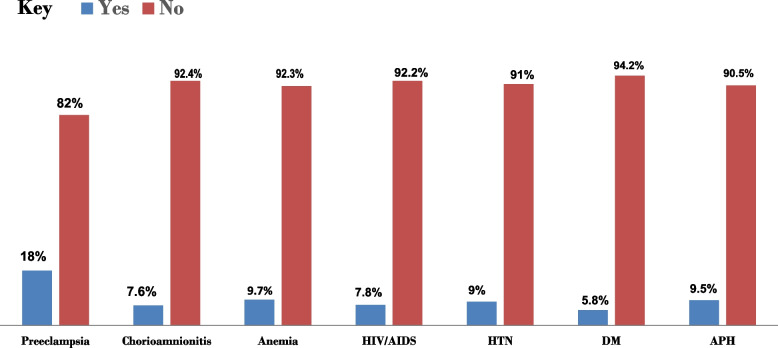
Maternal Complications in Preterm Neonate Cases. This figure presents the distribution of clinical and obstetric complications among mothers of preterm neonates admitted to the NICU of BHUTH and YGH from January 2021 to December 2022. The most common complications are Preeclampsia and premature rupture of membranes (PROM). The results highlight the significant burden of these complications on maternal health and the need for targeted interventions. Hypertension (HTN), Diabetes Mellitus (DM), and Antepartum Hemorrhage (APH)

The vast majority (83%) of mothers received antenatal care (Table 1). The median (IQR) age of mothers of the neonates was 30 (24-35) years. Most births were vaginal (63%) and singletons (81%). Deliveries primarily occurred at healthcare facilities (85%). Most mothers (76%) were multiparous (having given birth before). Figure 1 illustrates 42% of mothers have at least one form of medical or obstetrics complication during their current pregnancy. Preeclampsia (21%) and premature membrane ruptures (18%) were the most frequent complications.

**Table 1:** Clinical and obstetric characteristics of the mothers of preterm neonates admitted to NICU of BHUTH and YGH, 2023 from January 1, 2021-December 30, 2022 (*n* = 411)

Variables Category		Frequency	Percent (%)
Maternal age in years	≤ 19	37	9
	20–24	68	16.55
	25–29	96	23.36
	30–34	97	23.60
	≥ 35	113	27.49
Residence	Rural	169	41.12
	Urban	242	58.88
ANC follow up	Yes	343	83.21
	No	69	16.79
Number of ANC (*n* = 342)	1–3	306	69.3
	≥ 4	105	30.7
Mode of delivery	Spontaneous vaginal	260	66
	Cesarean section	134	28.87
	Instrumental	17	4.13
Place of delivery	At home	59	14.36
	Health institution	352	85.64
Institutional delivery (*n* = 352)	In born	255	72.44
	Out born	97	27.66
Type of current pregnancy	Singleton	333	81
	Multiple	78	20
Parity	Primiparous	100	24.33
	Multiparous	311	75.77
Gravidity	Primigravida	94	22.87
	Multigravida	277	67.40
	Grand multigravida	40	9.73

*ANC* Antenatal care, *BHUTH* Bule Hora University Teaching Hospital, *YGH* Yabelo General Hospital

#### Neonatal baseline characteristics and service delivery factors

**Fig. 2:**
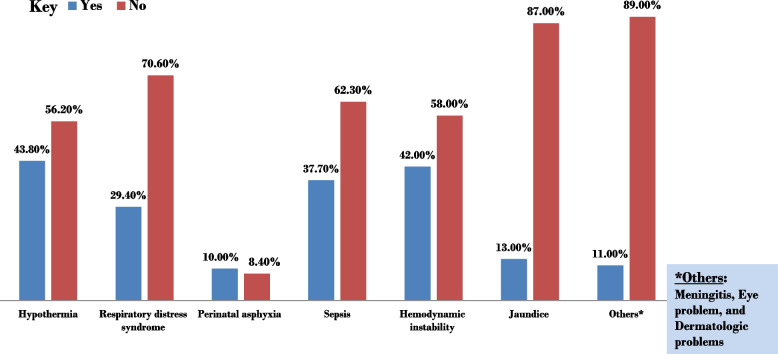
Neonatal Diagnoses in NICU. This figure illustrates the prevalence of various medical diagnoses among preterm neonates admitted to the NICU of BHUTH and YGH during the same period. Hypothermia and jaundice were identified as the highest and lowest complications occurred respectively. These findings underscore the critical healthcare needs of preterm infants and the importance of early intervention and specialized care

Both sexes were almost equally represented with a male to female ratio of 0.97 (Table 2). The median (IQR) weight of the neonates was 1600 (1400–2000) grams. Minimum and maximum gestational ages at birth were 28 and 36 weeks, respectively. Almost a quarter, 24.33% of the neonates had 1st minute Apgar score of less than 7. Additionally, 41.85% and 93.43% of admitted neonates were small for gestational age (SGA) and given antibiotics respectively. Regarding medical diagnosis, 92% of preterm neonates have at least one form of medical complication (Fig. 2). Hypothermia (43.80%) and jaundice (13.14%) were identified as the highest and lowest complications occurred respectively.

**Table 2:** Neonatal baseline characteristics and service delivery factors of preterm neonates admitted from January 2021-December 2022 to NICU of BHUTH and YGH, 2023(*n* = 411)

Variable	Category	Frequency	Percent (%)
Sex of the neonate	Female	208	50.61
	Male	203	49.39
Birth weight (BW)	< 2.5 kg	227	55.23
	< 1.5 kg	184	44.77
Gestational age (GA) in weeks	34 to < 37	185	45.01
	32 to < 34	121	29.44
	28 to < 32	105	25.55
1^st^minite APGAR score	< 7	100	24.33
	≥ 7	311	75.67
5th minute APGAR score	< 7	88	21.41
	≥ 7	323	78.59
Weight for gestational age	Small	172	41.85
	Appropriate	239	58.15
Kangaroo mother care	Yes	215	52.31
	No	196	47.69
Antibiotic	Yes	384	93.43
	No	27	6.57
Continuous Positive Airway Pressure (CPAP)	Yes	37	9
	No	374	91
Location of NICU neonates admitted	YGH	199	48.42
	BHUTH	212	51.58
Frequency of order revision (time duration between revision of treatment order by treating physician)	< 24 h	318	77.37
	> 24 h	93	22.63

### Time to initiate TF and overall survival function of preterm neonates

The overall incidence rate of TF was calculated using person-days of follow up as a denominator for the entire cohort. Accordingly, a total of 411 premature neonates were followed for a total of 690 neonate-days of observation with a minimum of 1 and maximum of 6 days. During the follow up period 301 (73.2%), 78 (19%), 28 (6.8%), and 4 (1%) premature neonates started TF, died, referred and left against medical advice, respectively.

Our study’s result indicates that the overall incidence density of TF initiation was reported as 43.6 per 100 neonate-days. Moreover, the median (IQR) time to initiate TF was found to be 2 (1–4) days. Additionally, in the first day (24 h), only 66 out of 411 neonates (16.1%) initiated TF (Table 3). By the end of 6 days (144 h), only 24 neonates remained, with 14 starting TF, 10 being censored, and the survival probability dropping to 0.0359 (95% CI: 0.01 to 0.074).

**Table 3:** The life table analysis of preterm neonates to start TF among preterm neonates admitted from January 2021-December, 2022 to NICU of BHUTH an YGH, 2023 (*n* = 411)

Time interval in days	Total beginning	Started Trophic Feeding	Censored	Survival probability	95%CI of survival probability
0–1	411	66	28	0.8338	(0.79, 0.87)
1–2	317	110	25	0.5326	(0.48, 0.58)
2–3	182	49	22	0.3800	(0.33, 0.43)
3–4	111	42	13	0.2272	(0.18, 0.27)
4–5	56	20	12	0.1363	(0.09, 0.18)
5–6	24	14	10	0.0359	(0.01, 0.074)

### Comparison of survivorship functions among different categorical variables

**Fig. 3:**
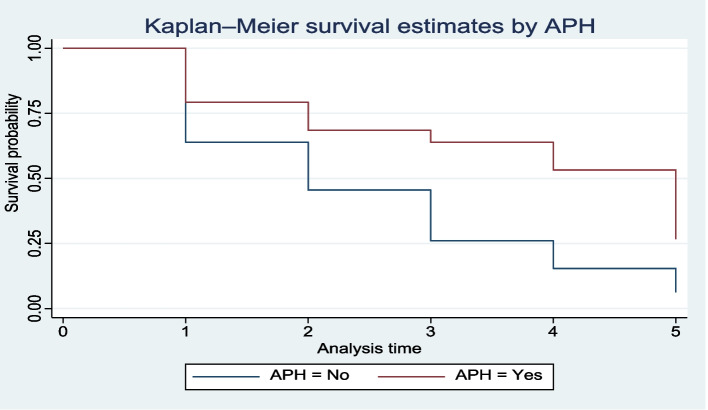
Survival Probability for TF Initiation Based on Maternal APH. This Kaplan–Meier curve depicts the survival probability of preterm neonates with and without maternal antepartum hemorrhage (APH) in terms of initiating total parenteral nutrition (TF). Preterm neonates born to mothers with APH exhibited a significantly later onset of TF compared to those born to mothers without APH. This finding highlights the potential impact of maternal complications on neonatal outcomes

**Fig. 4:**
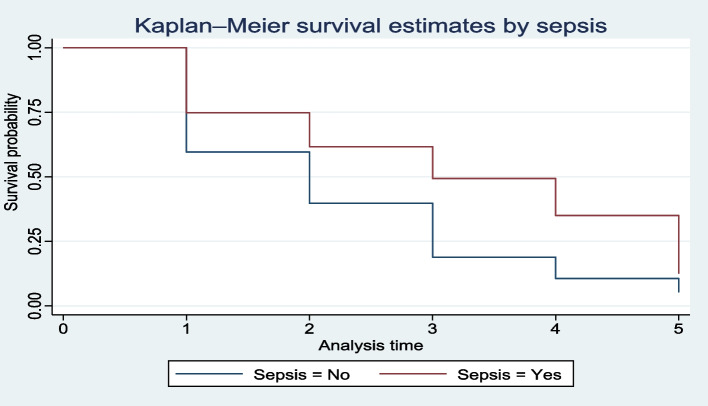
Survival for TF Initiation in Septic Preterm Neonates. This Kaplan–Meier curve examines the survival probability of preterm neonates with and without sepsis in relation to initiating TF. The analysis reveals that neonates with sepsis experienced a significantly delayed TF initiation compared to those without sepsis. These findings emphasize the critical role of early diagnosis and management of sepsis in improving neonatal outcomes

Kaplan–Meier curves (KMc) were constructed to compare the overall survival patterns over time for between different groups. Similarly, log-rank test was used in conjunction with KM curves to assess if the observed differences in survival between groups were statistically significant. Preterm neonates born to mothers with antepartum hemorrhage (APH) exhibited a significantly later onset of TF compared to those born to mothers without APH (Fig. 3). Likewise, the median time to TF commencement was statistically significant (log-rank test = 12.79, *P* < 0.001) (Table 4). Neonates with sepsis experienced a significantly delayed TF initiation compared to those without sepsis (Fig. 4). In a similar vein, Table 3 shows that a median time of 2 days for preterm neonates and 3 days for those with sepsis was significantly different (Log-rank test = 21.87, *P* < 0.001).

**Table 4:** Median survival time and log rank test of equality of survivorship to start TF among preterm neonates admitted from January 2021-December 2022 to NICU of BHUTH and YGH, 2023 (*n* = 411)

Variables		Median survival time in days (95% CI)	Log rank test **χ**^**2**^	*p-*value
PROM	Yes	3 (2, 4)	6.58	0.0103
	No	2 (2, 3)		
APH	Yes	5 (3, 6)	12.79	0.0003
	No	2 (2,3)		
Mode of delivery	SVD	2 (2, 2)	17.39	0.0001
	CS	3 (3, 4)		
Maternal HIV/AIDS	Yes	4 (2,)	8.46	0.0036
	No	2 (2, 3)		
Birth weight	< 1500	3 (2, 3)	5.48	0.0192
	≥ 1500	2 (2, 2)		
Gestational age	< 34	3 (2, 3)	7.79	0.0052
	≥ 34	2 (2, 2)		
Kangaroo mother care	Yes	2 (2, 2)	17.55	0.0001
	No	3 (2, 4)		
Hypothermia	Yes	3 (2, 3)	11.15	0.0008
	No	2 (1, 2)		
Sepsis	Yes	3 (3, 4)	21.87	0.0001
	No	2 (2, 2)		
Level of health care NICU located	2^0^ (YGH)	2 (1.5, 2.5)		
	3^0^ (BHUTRH)	3 (2.68, 3.31)		

*APH* Antepartum hemorrhage, *NICU* Neonatal Intensive Care Unit, *PROM* Premature rupture of membrane, *2°* Secondary, *3°* Tertiary

### Predictors of time to initiate trophic feeding among preterm neonates

In order to analyze the association between independent variables and time to initiate TF, first bi-variable cox regression was performed. Those variables with *p-*value of < 0.25 in bi-variable cox regression were entered into multivariable cox regression for adjusting the potential confounding variables. Accordingly, in Table 5, the identified significant predictors influencing the time to initiation of TF among preterm neonates at *p-*value of < 0.05 are displayed.

**Table 5:** Multivariable cox-proportional hazard regression on predictors of time to initiate TF among preterm neonates admitted from January 2021-December, 2022 to NICU of BHUTH and YGH, 2023 (*n* = 411)

Predictor variables		Trophic Feeding		CHR(95% CI)	AHR(95% CI)
		Started	Censored		
PROM	No	243	94	1.34 (1.003,1.79)	1.16 (0.85,1.57)
	Yes	58	16	1	1
Mode of delivery	Operative	92	59	1	1
	SVD	209	51	1.43(1.12, 1.83)	1.64 (1.26, 2.13)***
Preeclampsia	No	243	83	1.34 (1.005, 1.78)	1.27 (0.94, 1.72)
	Yes	58	27	1	1
APH	No	282	90	1.93 (1.21, 3.08)	1.63 (0.98, 2.71)
	Yes	19	20	1	1
Gestational age	34 to < 37	148	37	1	1
	32 to < 34	83	38	0.71 (0.54,0.93)	0.61 (0.46,0.81)**
	28 to < 32	70	35	0.77 (0.58, 1.02)	0.92 (0.67,1.25)
Birth weight (gram)	< 1500	113	71	0.77 (0.59, 1.00)	0.45 (0.34, 0.60)***
	≥ 1500	188	39	1	1
KMC	No	123	73	0.69(0.54, 0.87)	0.59 (0.46, 0.79)***
	Yes	178	37	1	1
Maternal HIV/AIDS	No	281	98	1.64 (1.04,2.59)	1.50 (0.94,2.39)
	Yes	20	12	1	1
Neonatal sepsis	No	208	48	1.58 (1.23, 2.02)	1.76 (1.36, 2.28)***
	Yes	93	62	1	1
Hypothermia	No	179	52	1.37 (1.09, 1.74)	1.51 (1.19, 1.93)**
	Yes	122	58	1	1
Level of hospital	2^0^ (YGH)	137	62	0.76 (0.61, 0.96)	0.78 (0.62, 0.99)*
	3^0^ (BHTH)	164	48	1	1

*CHR* crude hazard ratio, *AHR* adjusted hazard ratio, *SVD* Spontaneous vaginal delivery, *CS* Cesarean section, *APH* Antepartum hemorrhage, *WFGA* Weight for gestational age, *KMC* Kangaroo mother care, *CI* confidence interval, *2*^*0*^ secondary, *3*^*0*^ Tertiary

^*^statistically significant *p-*value < 0.05

^**^statistically significant *p-*value < 0.01

^***^statistically significant *p-*value < 0.001

Notably, neonates born vaginally were 64% more likely to initiate TF sooner compared to those born by cesarean section (AHR: 1.64, 95% CI: 1.26, 2.13). Preterm neonates between 32 and 34 weeks gestation had a delayed initiation of TF compared to their more mature counterparts (≥ 34 weeks) (AHR: 0.61, CI: 0.46, 0.81). Additionally, absence of kangaroo mother care was associated with a 41% reduced likelihood of earlier TF initiation (AHR: 0.59, CI: 0.46, 0.79). Likewise, very LBW (< 1500 g) preterm neonates had a 55% reduced chance of starting TF on time compared to those with > 1500 g (AHR: 0.45, CI: 0.34, 0.60). Interestingly, the absence of sepsis was also a significant predictor, with neonates without sepsis being 76% more likely to initiate TF compared to those with infection (AHR: 1.76, CI: 1.36, 2.28). Furthermore, the absence of hypothermia was associated with a 51% increased likelihood of earlier TF initiation (AHR: 1.51, CI: 1.19, 1.93). Finally, the level of hospital (YGH-level 2) was associated with a slight delay in initiating TF (AHR: 0.78, CI: 0.62, 0.99).

## Discussion

Our study’s objectives were to ascertain when TF was started and its determinants in preterm newborns hospitalized to Yabello General Hospital and Bule Hora University Teaching Hospital’s NICU between January 1 2021 and December 30 2022. As a result, of the 411 neonates observed on the first day of follow-up, 66 began TF, and 28 were censored. However, only 24 were left at the end of the five-day follow-up, with 14 of them having started TF. The overall incidence density in our sample was 43.6 per 100 neonate days. This means, out of every 100 days that newborn babies were observed in our study, TF was started in an average of 43.6 of those days. The median (IQR) time to TF onset was 2 (1–4) days. Moreover, the study revealed a significant increase in preterm neonatal mortality in Ethiopia, with 113.04 deaths per 1000 neonate-days, a significant increase from previous research. GA (> 34 weeks), Spontaneous Vaginal delivery (SVD), the absence of sepsis, and the absence of hypothermia increase the probability of early initiation of TF by 64%, 76%, and 51%, respectively. Conversely, it was discovered that delayed TF start was caused by BW (< 1500 g), lack of kangaroo mother care, and being admitted to a level-2 hospital.

In our study, we found that only 21.93% of preterm neonates initiated feeding within the first 24 h of birth, indicating that many did not start within this optimal timeframe. The study’s findings are lower than previous studies in Portugal 44% [43], Tuscany Italy (74.1%) [44], Iran (36%) [45], Nigeria and Kenya 48% [30], New Zealand (60%) [25], Addis Ababa (29.9%) [34] and Amhara, Northwest Ethiopia (24.76%) [21]. The discrepancy may be due to differences in service quality, and availability of specialized facilities, milk banks, study period length, sample size, and design between the studies. Moreover, nearly half, (46.74%) of preterm neonates initiated TF within 48 h of birth. This is comparable with a study conducted in Addis Ababa, Ethiopia (48.2%) [34]. However, it is lower than the findings from Nigerian Special Care Baby Unit (66.7%) [46], Iran (63.2%) [45], Uganda rural hospitals (80%) [32] and New Zealand (80%) [25]. This highlights a significant delay in initiating TF among preterm neonates and creates concerns about high neonatal mortality as evidenced by our study where, over 50% of the neonates died within the first 48 h of life. This could be because of their initial treacherous condition. The result of this study regarding incidence density of TF is lower than the result of a follow up study conducted in northern Ethiopia [21], where the incidence density of starting TF was 48 per 100 neonate-days. Moreover, the median time of starting TF of 2 (IQR: 1–4) days, found in our study, is slightly higher than the result of a follow up study conducted in China [47] and a retrospective study in New Zealand [25], which had a similar median time of 1 day. This discrepancy might be due to difference in service delivery quality, hospital level, having well-equipped institution, sample size and study settings. However, our result is consistent with a longitudinal study in Ethiopia in which median time to start TF was less than 2 days [21] and a multicenter observational follow up study in Nigeria and Kenya [37], which might be due to similarity in study design and characteristics of study subjects. This implies the presence of a significant delay to initiate TF for studied preterm neonates.

Preterm neonatal mortality (NMR) was found to be surprisingly common in our study. We found an astounding 113.04 deaths per 1000 neonate-days, a significant increase above earlier research done in various parts of Ethiopia. From 27 per 1000 neonate-days in southern Ethiopia [48], 29.438 per 1000 neonate-days in Addis Ababa, Central Ethiopia [49] and to 75.63 per 1000 neonate-days in northwestern Ethiopia [50], these previous investigations revealed substantially lower fatality rates. The NMR of 45.15 per 1000 neonate-days was reported in a study conducted in Hawassa City, Ethiopia [27], which is geographically similar to our study area. Even the neighboring African country, Burkina Faso had a much lower rate, 1.93 deaths per 1000 person-days [51]. The study’s unexpected high NMR result led to a potential explanation of a low proportion of neonates initiating TF early in their lives. Previous research suggests that preterm neonates who do not receive TF early are more susceptible to death [49]. Studies have shown that early TF initiation offers several benefits – it reduces the length of hospital stays, lowers infection rates, and promotes gut development [24, 52, 53]. Conversely, a delay in starting TF might hinder the maturation of the gut, leading to a weakened immune system [54, 55]. This, in turn, could significantly increase the risk of complications like NEC and various infections, ultimately contributing to higher neonatal mortality [56]. Therefore, we strongly suggest that, in particular, for susceptible groups such as preterm newborns, targeted interventions encouraging early TF initiation could drastically lower NMR rates and increase survival rates.

In this study, the chance of starting TF on time was 55% reduced among very low birth weight (< 1500 g) preterm neonates when compared to those with a birth weight of > 1500 g. This finding is in line with the results of retrospective longitudinal a study in China [47], a retrospective study in New Zealand [25] and a multicenter prospective study conducted in Ethiopia [21]. This might be due to perceived under-development of organs and low readiness to enteral feeding of preterm neonates with lower birth weight. However in contrast to this, guidelines on feeding of very low birth weight infants recommend early initiation of TF within 1 day after birth, also for this group of preterm neonates [11]. This result highlights the importance of individualized care plans based on birth weight in order to insure early TF for VLBW preterm neonates.

Among preterm neonates with a gestational age of less than 34 weeks, there was a statistically significant 39% reduction in the likelihood of initiating TF in comparison to neonates with a gestational age of 34 weeks or more. This result is consistent with findings of observational prospective follow up study in Italy [44], retrospective follow up study in China [47], a cross sectional study carried out in two of African countries [30], a multicenter prospective study conducted in Ethiopia [20], prospective follow up study conducted in northern Ethiopia [21] and cross sectional study in Spain [17], which revealed delayed commencement of TF among newborns with lower gestational age. This delay may be attributed to factors such as incomplete intestinal maturation, feeding intolerance, and perceived risk of NEC. Preterm neonates born before 34 weeks of pregnancy may experience gastrointestinal issues. Early identification and support of lower gestational age preterm neonates may facilitate timely initiation of TF in this vulnerable group.

The study found that preterm neonates delivered through spontaneous vaginal delivery had a 64% higher likelihood of starting TF compared to those delivered through CS, possibly due to delayed breast milk provision. The study’s findings align with a multicenter prospective follow-up conducted in northern Ethiopia [21], prospective follow-up conducted in Addis Ababa, Ethiopia [34], and Italy [29]. This might be because of the reason that newborn’s post-delivery needs may require additional monitoring and assistance, while the mother’s recovery from the surgery may take longer. The result implies that preterm neonates delivered with CS are at risk of experiencing delayed initiation of TF and related complications. Thus, the healthcare providers must closely monitor both mothers and babies post-delivery and initiate enteral feeding as soon as the baby is stable and ready. However, this finding is in contrast with a result of observational retrospective follow up study conducted in China which revealed lack of significant association of delivery mode and time to initiate enteral feeding [47]. This discrepancy might be due to difference in characteristics of studied subjects, sample size and study setup.

WHO recommended KMC for preterm or low-birth-weight infants, starting in healthcare facilities or at home, and lasting 8–24 h daily [57]. In line with this evidence, in our study, absence of KMC was associated with a 41% reduced likelihood of earlier TF initiation as compared to those with the care. Similar to this finding, a retrospective follow up study in Turkiye has identified KMC as a main factor to improve enteral feeding skills among preterm neonates [58]. Moreover, a prospective cohort study done in a teaching hospital in India identified that early KMC was safe and associated with reduced time to full feeds (TFF) in preterm neonates [59]. A meta-analysis of randomized controlled trial studies reported that KMC encourages early breastfeeding initiation among preterm and low birth weight infants [60]. This implies that KMC is a gentle, effective method for preterm infants, promoting their health and well-being by allowing early discharge and avoiding agitation in busy wards [61]. On the other hand, preterm neonates without hypothermia had a 51% higher hazard of starting TF when compared to those with hypothermia. This is line with a study conducted in Kuala Lumpur Maternity Hospital [62]. This might be due to the fact that most preterm neonates with hypothermia often receive radiant warmer treatment and stay separated from mothers, making early enteral feeding unsuitable. This implies the significance of effective temperature regulation and thermal management strategies in the care of preterm neonates to facilitate timely initiation of TF.

In this study, the hazard of starting TF was 76% increased among premature neonates without sepsis as compared to those with sepsis. This finding is supported with a result of a retrospective study conducted in Kaplan medical center, Israel [63], Addis Ababa, Ethiopia [34], and Maharashtra, India [64]. This similarity might be due to the fact that sepsis can cause decreased gut motility, increased risk of NEC [65], and damage the gut lining [66], making it difficult for neonates to tolerate enteral feeding. Thus, the healthcare providers may delay TF initiation to control infection, reduce NEC risk, and minimize gut permeability. However in contrast to this, guideline on TF does not consider sepsis as contraindication of TF among premature infants and recommends enteral feeding to be initiated early for this group of preterm infants [11]. The result implies early prevention and treatment of sepsis among preterm neonates might support timely initiation of TF.

Our study revealed inconsistency in how TF is initiated for preterm neonates across different healthcare levels. We observed a surprising trend – neonates admitted to the secondary-level hospital (YGH) were 22% less likely to receive TF compared to those in the tertiary hospital (BHTRH). This finding is particularly concerning, as timely initiation of TF is crucial for the survival and development of preterm infants. Similar trends were recognized in prior research conducted in Addis Ababa, Ethiopia [34], highlighting a potential issue within the Ethiopian healthcare system. Additionally, studies across geographically distinct locations like Nigeria and Kenya have reported similar disparities [30, 37]. This observed similarities suggests a concerning possibility that preterm neonates in less-resourced facilities may face systemic disadvantages when it comes to accessing essential and life-saving interventions like TF. Moreover, the secondary hospitals might have lack of resources, trained personnel, and logistical challenges. A study on pediatrics’ social role reveals a shift from curative to comprehensive approach, emphasizing holistic competencies to address children’s complex health needs, including biological, cultural, and psychological factors [67]. Therefore, our study highlights the need for a comprehensive investigation into the disparity in access to TF across healthcare levels, including capacity building, resource allocation, and logistical processes.

To the best of our knowledge our study is among few in Ethiopia conducted to determine the time to initiate trophic feeding, involving advanced statistical analysis. However, due to retrospective nature, incomplete data was excluded, potentially introducing selection bias. The data was collected from secondary sources, potentially missing important predictors like nurse-patient ratio and breast milk availability.

## Conclusion

Our study highlights significant opportunities to improve early trophic feeding (TF) practices for preterm neonates**.** Only 16.6% initiated TF within the first 24 h, suggesting a gap between current practice and optimal timing. Additionally, disparities exist across healthcare levels, with neonates in secondary-level hospitals 22% less likely to receive TF compared to those in tertiary centers. Factors promoting early TF initiation include: Vaginal delivery (SVD), Absence of sepsis or hypothermia and Kangaroo mother care. Conversely, early TF initiation is hindered by: Lower gestational age (32–34 weeks), Lower birth weight (< 1500 g), Lack of kangaroo mother care and Admission to a secondary hospital. Based on these findings, we propose the following feasible and practical recommendations: Implement clear guidelines for initiating TF based on gestational age, birth weight, and clinical condition, ensuring consistency across healthcare levels. Provide training and resources to healthcare professionals at secondary facilities to ensure they feel confident initiating TF for eligible preterm neonates. Encourage and support kangaroo mother care practices for all eligible preterm neonates, recognizing its positive impact on TF initiation. Ensure adequate staffing and equipment’s are available at both secondary and tertiary hospitals to support early TF implementation.

## Supplementary Information

Supplementary Material 1.

Supplementary Material 2.

## Data Availability

For those who are interested; the datasets of this study could be accessed from the corresponding author on reasonable request.
