# Supplementary material for: How long does it take to start minimal enteral feeding in preterm Neonates admitted to NICUs in Southern Oromia, Ethiopia?
Source: Ital J Pediatr. 2025 Feb 7;51:27. doi: 10.1186/s13052-025-01876-1 (PMC11803979; doi:10.1186/s13052-025-01876-1)
Supplement: Supplementary file 1 — Supplementary Material 1. [file 13052_2025_1876_MOESM1_ESM.docx]

**Supplementary Figure 1a:** **−ln(−ln) survival plot**

**Supplementary Figure 1b:** **−ln(−ln) survival plot**

**Supplementary Figure 1c:** **−ln(−ln) survival plot**

**Supplementary Figure 1d:** **−ln(−ln) survival plot**

**Supplementary Figure 1e:** **−ln(−ln) survival plot**

**Supplementary Figure 2a:** **Kaplan Meier and predicted survival plot**

**Supplementary Figure 2b:** **Kaplan Meier and predicted survival plot**

**Supplementary Figure 2c:** **Kaplan Meier and predicted survival plot**

**Supplementary Figure 2d:** **Kaplan Meier and predicted survival plot**

**Supplementary Figure 2e:** **Kaplan Meier and predicted survival plot**

**Supplementary Figure 2f:** **Kaplan Meier and predicted survival plot**
