# Supplementary material for: How long does it take to start minimal enteral feeding in preterm Neonates admitted to NICUs in Southern Oromia, Ethiopia?
Source: Ital J Pediatr. 2025 Feb 7;51:27. doi: 10.1186/s13052-025-01876-1 (PMC11803979; doi:10.1186/s13052-025-01876-1)
Supplement: Supplementary file 2 — Supplementary Material 2. [file 13052_2025_1876_MOESM2_ESM.docx]

**Supplementary Table 1:** **Cox Proportional hazard assumption****: Schoenfeld residual test to assess proportional hazard assumption**

| Covariates | rho | chi2(x^2^) | Prob>chi2 (p-value) |
| --- | --- | --- | --- |
| Delivery mode | 0.00972 | 0.02 | 0.8820 |
| PROM | -0.01793 | 0.07 | 0.7846 |
| Maternal HIV/AIDS | 0.04801 | 0.52 | 0.4714 |
| Maternal anemia | -0.02566 | 0.14 | 0.7047 |
| Sepsis | 0.00293 | 0.00 | 0.9655 |
| Hypothermia | 0.06749 | 1.01 | 0.3142 |
| Preeclampsia | 0.05052 | 0.62 | 0.4317 |
| APH | -0.02115 | 0.10 | 0.7491 |
| KMC | 0.00291 | 0.00 | 0.9667 |
| Gestational age | -0.05635 | 0.71 | 0.3981 |
| Birth weight | -0.02004 | 0.08 | 0.7807 |
| **Global test** | | 3.84 | 0.9743 |
